# Word-Blindness with Unusual Features

**Published:** 1891-07

**Authors:** 


### Word-Blindness with Unusual Features.

The following case was reported by Professor Mierzejewski at a recent meeting of the St. Petersburg Society of Psychiatry, and is given in the *Neurologisches Centralblatt* for December 15, 1890.

A physician, fifty-six years old, had had syphilis in his youth, and for several years had suffered from chronic nephritis. In January, 1890, he had an attack of uræmic coma, which lasted four or five days. He had since had two other attacks of shorter duration. Some time after the third attack, which occurred last spring, the patient noticed that he had lost the power of reading, although he could distinguish the letters easily, and his sight in general was unchanged.

Mierzejewski found the following on examination: The patient sees each individual letter clearly, but is unable to join the letters into syllables or words. He writes without difficulty and correctly whatever is dictated to him, but can not read what he had written. He can write prescriptions in due form but can not read them afterward. He can make correct copies without understanding the meaning of the words copied. Numbers, however, he can read and pronounce correctly. The patient's sight is perfect, and the fundus of the eye is normal. There is no disturbance of speech and the intelligence is unaffected. There is no change in sensation, motion, or the reflexes. After looking through the literature of the subject, Mierzejewski concludes that this is the first case of word-blindness as yet reported in which the ability to distinguish the single letters was retained, and he calls it *cœcitas syllabaris et verbalis, sed non litteralis.*—*N. Y. Med. Jour.*

